# Role of aortic stiffness and inflammation in the etiology of young-onset hypertension

**DOI:** 10.3906/sag-1908-137

**Published:** 2019-12-16

**Authors:** Serkan GÖKASLAN, Çiğdem ÖZER GÖKASLAN, Emin DEMİREL, Sefa ÇELİK

**Affiliations:** 1 Department of Cardiology, Faculty of Medicine, Afyonkarahisar University of Health Sciences, Afyonkarahisar Turkey; 2 Department of Radiology, Faculty of Medicine, Afyonkarahisar University of Health Sciences, Afyonkarahisar Turkey; 3 Department of Biochemistry, Faculty of Medicine, Afyonkarahisar University of Health Sciences, Afyonkarahisar Turkey

**Keywords:** Aortic stiffness, young-onset hypertension, inflammation markers

## Abstract

**Background/aim:**

Young-onset hypertension is a form of condition diagnosed in patients aged below 40. Cytokines such as interleukin (IL)-6 and also MCP-1 may play a role in the development of arterial hypertension. Aortic stiffness can be detected by measuring pulse wave velocity (PWV). We aimed to explore the relationship between inflammation and aortic stiffness and investigate their roles in the etiology of young-onset hypertension.

**Materials and methods:**

We enrolled 16 patients diagnosed with young-onset hypertension and 16 volunteers without hypertension. The plasma levels of MCP-1 and IL-6 were determined using an enzyme-linked immunosorbent assay and quantitative enzyme-linked immunoassay, respectively. Carotid-femoral PWV was measured using an arteriograph device.

**Results:**

Compared with those in normotensive controls, the plasma levels of IL-6 and MCP-1 and the PWV values were significantly higher in patients with young-onset hypertension (P < 0.001). PWV values were also positively correlated with the levels of MCP-1 and IL-6. However, no statistically significant difference was noted in intima-media thickness ​​between the two groups (P = 0.224).

**Conclusion:**

In this study, increased PWVs and the levels of inflammation markers were associated with aortic stiffness and inflammation in patients with young-onset hypertension, suggesting they have a role in the etiology of hypertension.

## 1. Introduction

Hypertension is a major cause of mortality worldwide [1]. Young-onset hypertension is a form of this condition diagnosed in patients aged below 40. In an epidemiological study, the incidence of young-onset hypertension was approximately 0.1%, and sex, genetics, and obesity were the risk factors [2]. The predominant form of hypertension in individuals aged below 50 is essential hypertension, while isolated systolic hypertension is more commonly seen among elderly individuals [3,4].

Experimental evidence suggests that hypertension functions to aggravate inflammation by promoting the expression of cytokines [5,6]. A study conducted on healthy men demonstrated a correlation between increased blood pressure (hypertension) and elevated levels of circulating interleukin (IL)-6 [7]. Increase in IL-6 was also reported in response to the infusion of angiotensin II in humans, demonstrating a direct relationship between the two [8,9]. In addition to IL-6, MCP-1, a key chemokine involved in the onset of inflammation, may play a role not only in various pathophysiological processes occurring in the cardiovascular system but also in the development of arterial hypertension [10,11]. Vascular endothelial cells stimulate the expression of MCP-1 by mediating the inflammatory cytokines IL-1, IL-4, IL-6, and tumor necrosis factor-α [12]. 

Aortic stiffness contributes to vascular diseases by inducing vascular strain and endothelial dysfunction. Moreover, pulse wave velocity (PWV) has been widely accepted for diagnosing aortic stiffness in clinical practices [13–15]. Carotid-femoral PWV is recommended by the European Society of Hypertension, the European Society of Cardiology, and the American Heart Association as a clinical marker for the classification of cardiovascular risk in patients with hypertension [16–18]. The carotid intima-media thickness (IMT) is also widely used for detecting early atherosclerosis and is associated with increased cardiovascular risk factors, such as hypertension, diabetes mellitus, hypercholesterolemia, obesity, and coronary artery disease [19–22]. 

However, to date, the etiology of young-onset hypertension has been unclear. In this study, we aimed to explore the relationship between inflammation and aortic stiffness and investigate their roles in the etiology of young-onset hypertension.

## 2. Materials and methods

### 2.1. Patients

Between December 2017 and May 2018, 16 patients diagnosed with young-onset hypertension (patient group) and 16 volunteers (control group) without hypertension (all aged below 40) were included in the study. Their sedimentation rates, C-reactive protein levels, and white blood cell levels ​​were within normal limits. Patients with rheumatic disease, hyperlipidemia, and acute or chronic infections were excluded. Both groups were matched for age and sex. Informed consent was obtained from all participants, and the study was approved by the ethics committee of the Faculty of Medicine of Afyonkarahisar University of Health Sciences. 

### 2.2. Enzyme-linked immunosorbent assay (ELISA) 

The plasma levels of MCP-1 were determined using an ELISA kit (BMS281TEN; eBioscience, Vienna, Austria) as per the manufacturer’s recommendations. Absorbance was measured at 450 nm using a spectrophotometer (BioTek, Epoch Microplate Spectrophotometer, Winooski, VT, USA). The limit of detection and coefficient of interassay variation were 2.31 pg/mL and 8.7%, respectively. The level of IL-6 was measured using a quantitative enzyme-linked immunoassay kit (Elabscience, Houston, TX, USA).

### 2.3. Determination of carotid-femoral PWV

Carotid-femoral PWV was measured using an arteriograph device (TensioMed, Budapest, Hungary). The pulse waveforms of the common carotid and femoral arteries were sequentially acquired. Subsequently, for determining PWV, the distance from the suprasternal notch to the femoral artery was divided by the time interval between the waves.

### 2.4. IMT measurements 

The IMT of the right and left common carotid arteries was measured from the distant walls based on the Mannheim carotid IMT consensus [23]. An Aplio MX duplex Doppler ultrasonography device (Toshiba, Otawara, Tochigi, Japan) and a 7.5-MHz probe were used to obtain images of the intima media of the carotid artery. The best image was acquired when the patient was in the supine position. After obtaining the mean and maximum IMTs of the distal walls of the right and left arteries at 1–2 cm proximally to the bulb, the average of the mean values ​​of the two measurements was calculated.

### 2.5. Statistical analysis

All data were analyzed using SPSS 21.0 (IBM Corp., Armonk, NY, USA). Comparison of the categorical and continuous variables was performed using the chi-square and Mann–Whitney U tests, respectively. The association between MCP-1, IL-6, and PWV was investigated with Spearman’s correlation coefficient. An a priori power analysis could not be conducted owing to the unavailability of pilot data. Therefore, a post hoc power analysis was conducted to confirm that the current study had adequate power (99%).

## 3. Results

Participant demographics are shown in Table 1. Among 16 patients with young-onset hypertension who participated in the study, nine were males and seven were females, with a mean age of 31.6 (range: 18–40) years. The control group comprised 16 individuals, eight males and eight females, with a mean age of 29.9 (range: 18–39) years. The body mass index (BMI) and the mean levels of low-density lipoprotein (LDL) and triglycerides of the two groups are shown in Table 1. There were no significant differences in any of the variables examined between the patient and the control groups. 

**Table 1:** The demographic features of patients with young-onset hypertension and normotensive controls.

	Patients (n = 16) (mean ± SD)	Controls (n = 16) (mean ± SD)	P-value
Age (mean)	31.6 ± 8.5	29.9 ± 5.9	0.642
Sex (male)	9 (56%)	8 (50%)	0.469
BMI (kg/m2)	27.49 ± 3.51	24.61 ± 4.36	0.094
LDL (mg/dL)	95.8 ± 18.4	97.2 ± 20.6	0.128
Triglyceride (mg/dL)	183.2 ± 25.6	178.5 ± 23.2	0.076

The mean level of IL-6 significantly differed between the patient (152.45 ± 80.93; range: 52.96–317.08 pg/mL) and the control groups (5.73 ± 5.94; range: 1.02–25.53 pg/mL; P < 0.001). The level of MCP-1 was significantly higher in the patient group (293.15 ± 148.76; range: 34.55–614.63 pg/mL) than that in the control group (13.49 ± 41.38; range: 0.39 ± 168.42 pg/mL; P < 0.001). 

Mean PWV in the patient group (11.22 ± 2.54; range: 7.5–16 m/s) was significantly higher than that of the control group (7.36 ± 1.86; range: 4.6–10.2 m/s; P < 0.001). PWV was positively correlated with the levels of MCP-1 and IL-6. PWV level was positively correlated with IL-6 (r = 0.656, P <0.001) and MCP-1 (r = 0.614, P <0.001).

Mean IMT in the patient group (0.57 ± 0.14; range: 0.3–0.9 mm) was not significantly different from that in the control group (0.5 ± 0.1; range: 0.3–0.7 mm; P = 0.224) (Table 2).

**Table 2:** Mean levels of IL-6, MCP-1, PWV, and IMT of patients with young-onset hypertension and normotensive controls.

	Patients (mean ± SD)	Controls (mean ± SD)	P-value
IL-6 (pg/mL)	152.45 ± 80.93	5.73 ± 5.94	<0.001
MCP-1(pg/mL)	293.15 ± 148.76	13.49 ± 41.38	<0.001
PWV (m/s)	11.22 ± 2.54	7.36 ± 1.86	<0.001
IMT (mm)	0.57 ± 0.14	0.5 ± 0.1	0.224

## 4. Discussion

To the best of our knowledge, this is the first study to demonstrate that patients with young-onset hypertension have significantly higher levels of plasma MCP-1 and IL-6 and aortic stiffness than normotensive individuals. These results support the use of IL-6 and MCP-1 as the biological markers of vascular impairment.

The earlier the onset of hypertension is, the longer the exposure time to the disease and the higher the risk of cardiovascular events will be. Therefore, appropriately identifying and treating the underlying pathogenesis of young-onset hypertension is important to prevent cardiovascular events. However, the literature on this subject is not sufficient. A study involving young adults demonstrated that the main hemodynamic abnormality underlying essential hypertension may be increased peripheral vascular resistance, which causes vascular remodeling in the arteries, involving extracellular matrix deposition and inflammation [24]. 

Previous studies showed that vascular inflammation caused vascular damage and played a key role in the pathogenesis and progression of hypertension [25–27]. However, the role of inflammatory cytokines in the mechanism and progression of hypertension remains unclear. The stimulation of human vascular smooth muscle cells by angiotensin II, the main regulator of blood pressure, resulted in increased expression and release of IL-6 [28–30]. IL-6 increased vascular smooth muscle cell proliferation, which is a characteristic of the early stages of hypertension [31]. Additionally, MCP-1 also contributed to the onset of inflammation by promoting the uptake of inflammatory cells into the vessel wall [32]. Furthermore, increased blood vessel tension due to high blood pressure increased the expression of MCP-1 mRNA in human vascular endothelial cells, which further potentiated the secretion of MCP-1 [33,34]. Thus, in young-onset hypertension, elevated levels of MCP-1 and IL-6 in the vascular endothelium may indicate the stimulation of cellular immunological processes that contribute to early vascular aging and the development of hypertension [35,36].

The present study provides evidence regarding high levels of IL-6 and MCP-1 and a predisposition to inflammation in patients with young-onset hypertension compared with those in normotensive individuals. These mechanisms may partly explain the relationship observed between increased levels of blood pressure, MCP-1, and IL-6, suggesting that IL-6 and MCP-1 may serve as the biological markers of vascular impairment.

Another known independent predictor of cardiovascular disease is increased aortic stiffness, which is considered to be an important cardiovascular risk factor [37,38]. Although increased aortic stiffness can be determined by measuring pulse waves, the latter is an independent predictor of poor cardiovascular outcomes in patients with essential hypertension [24,39]. A metaanalysis found that a 1-m/s increase in PWV was associated with an 11% increase in cardiovascular deaths [37].

Carotid-femoral and brachial-ankle PWVs are the two most commonly used PWV measurements. Brachial-ankle PWV calculation involves both elastic and muscular arteries, and is considered to be a predictor of aortic stiffness. Conversely, carotid-femoral PWV, which only involves measurement of the elastic artery, is accepted as a better indicator [17]. Aortic PWV increases with age and accelerates with the presence and severity of hypertension [40,41]. In a study using carotid-femoral PWVs, hypertension was associated with the progression of aortic stiffness in young patients compared with those in normotensive subjects [42]. The findings of this study are in concordance with those in the literature. Carotid-femoral PWV increased in patients with young-onset hypertension compared with those in the control group. This suggests that in addition to predisposition to inflammation, increased aortic stiffness contributes to the etiology of young-onset hypertension. Furthermore, the positive relationship between PWVs and the levels of MCP-1 and IL-6 indicates that aortic stiffness may play a role in the etiology of young-onset hypertension. 

Most young individuals with hypertension show early vascular changes despite short-term exposure to the disease [43]. As increased carotid IMT is strongly associated with the early stages of vascular atherosclerosis in young adults, this measure can be used to evaluate early atherosclerosis and predict cardiovascular events [43]. However, in this study, we found no significant increase in carotid IMT or the evidence of early atherosclerosis. Reportedly, an increase in carotid IMT is adaptive to medial hypertrophy rather than intimal hypertrophy in young patients with hypertension [43–45]. In our study, the lack of difference in the carotid IMTs ​​in the patient group compared with the difference observed in the control group may be attributed to the small media of the carotid artery and the inability to radiologically detect medial hypertrophy [46,47]. In addition, the patients included in this study were young; accordingly, atherosclerosis is not expected in this age group, which may be the reason why no significant difference was observed between the two groups regarding IMT. However, this was strictly a clinical study, and additional histopathological studies are warranted to confirm the findings. The other limitation of this study was that the patient group was not compared with hypertensive patients over 40 years of age. Such a comparison could increase the effectiveness of the study.

In conclusion, increased PWV and the levels of inflammatory markers were associated with aortic stiffness and inflammation in patients with young-onset hypertension, suggesting that these factors have a role in the etiology of hypertension.
